# Supplementary material for: New Quinolone-Based Thiosemicarbazones Showing Activity Against Plasmodium falciparum and Mycobacterium tuberculosis
Source: Molecules. 2019 May 4;24(9):1740. doi: 10.3390/molecules24091740 (PMC6540015; doi:10.3390/molecules24091740)

Supplementary Materials: New Quinolone-Based Thiosemicarbazones Showing Activity Against *Plasmodium falciparum* and *Mycobacterium tuberculosis*

Richard M. Beteck[1]*, Ronnett Seldon[2], Audrey Jordaan[3], Digby F. Warner[3,4,5], Heinrich C. Hoppe[6,7], Michelle Isaacs[7], Dustin Laming[7], and Setshaba D. Khanye[1,7]*

[1] Faculty of Pharmacy, Division of Pharmaceutical Chemistry, Rhodes University, Grahamstown 6140, South Africa

[2] Drug Discovery and Development Centre (H3-D), Department of Chemistry, University of Cape Town, Rondebosch 7701, South Africa

[3] SAMRC/NHLS/UCT Molecular Mycobacteriology Research Unit, Department of Pathology, University of Cape Town, Observatory 7925, South Africa

[4] Institute of Infectious Disease and Molecular Medicine, University of Cape Town, Observatory 7925, South Africa

[5] Wellcome Centre for Infectious Diseases Research in Africa, University of Cape Town, Observatory 7925, South Africa

[6] Faculty of Science, Department of Biochemistry and Microbiology, Rhodes University, Grahamstown 6140, South Africa

[7] Centre for Chemico- and Biomedicinal Research, Rhodes University, Grahamstown 6140, South Africa

* Correspondence: s.khanye@ru.ac.za (S.D.K); Tel.: +27-46-603-8397 (S.D.K)

Received: date; Accepted: date; Published: date

COMPOUND 11

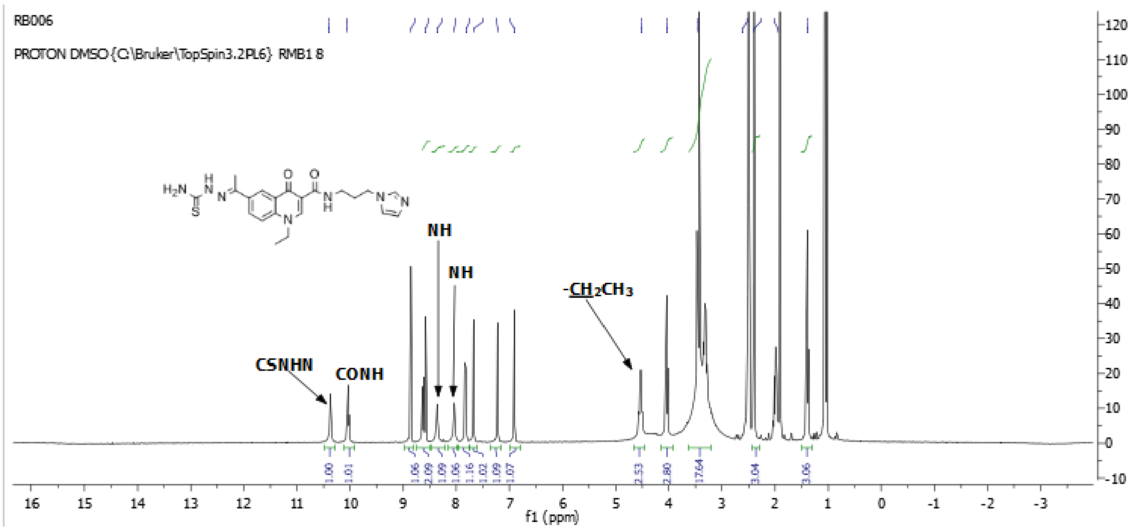
RB006
PROTON DMSO {C:\Bruker\TopSpin3.2PL6} RMB1 8
CSNHN
CONH
NH
NH
-CH2CH3
1.00
1.01
1.06
2.09
1.09
1.06
1.16
1.02
1.09
1.07
2.53
2.80
17.64
3.04
3.06
f1 (ppm)

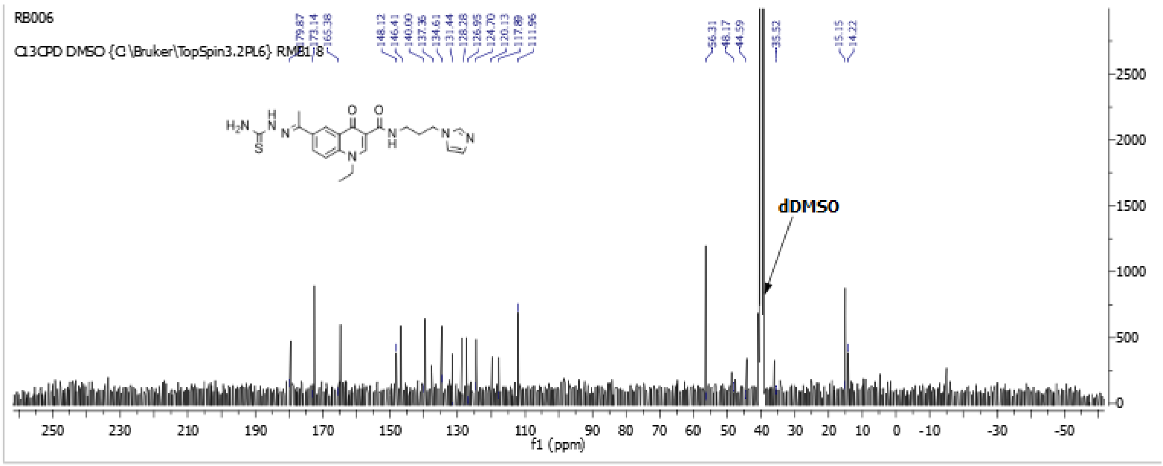
RB006
C13CPD DMSO {C:\Bruker\TopSpin3.2PL6} RMB1 8
179.87
173.14
165.38
148.12
146.41
140.00
137.36
134.61
131.44
128.28
126.95
124.70
120.13
117.89
111.96
56.31
48.17
44.59
35.52
15.15
14.22
dDMSO
f1 (ppm)

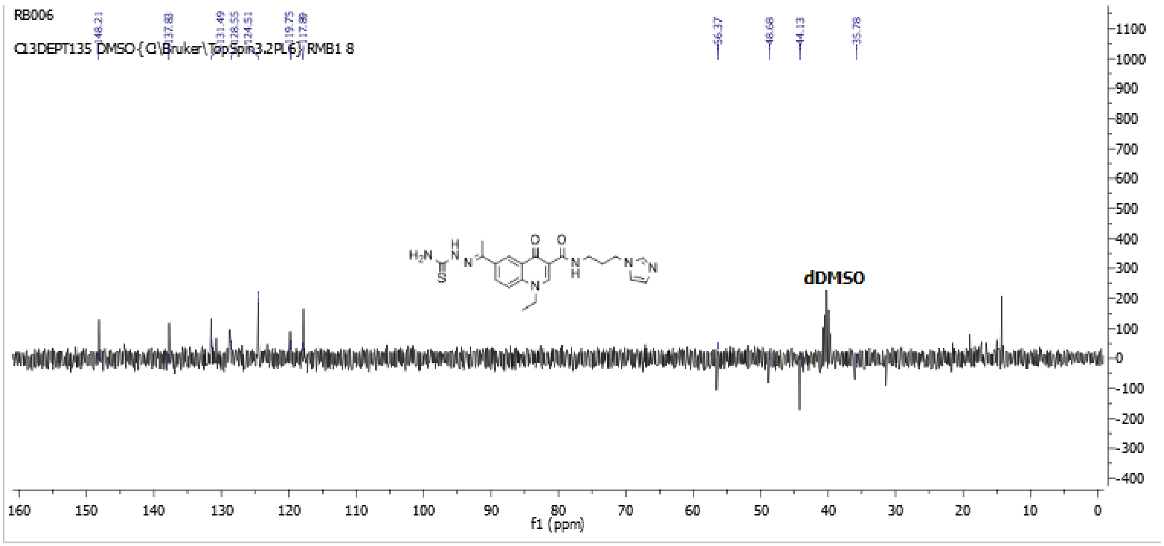
RB006
C13DEPT135 DMSO {C:\Bruker\TopSpin3.2PL6} RMB1 8
148.21
137.83
131.49
128.55
124.51
119.75
117.89
56.37
48.68
44.13
35.78
dDMSO
f1 (ppm)

COMPOUND 12

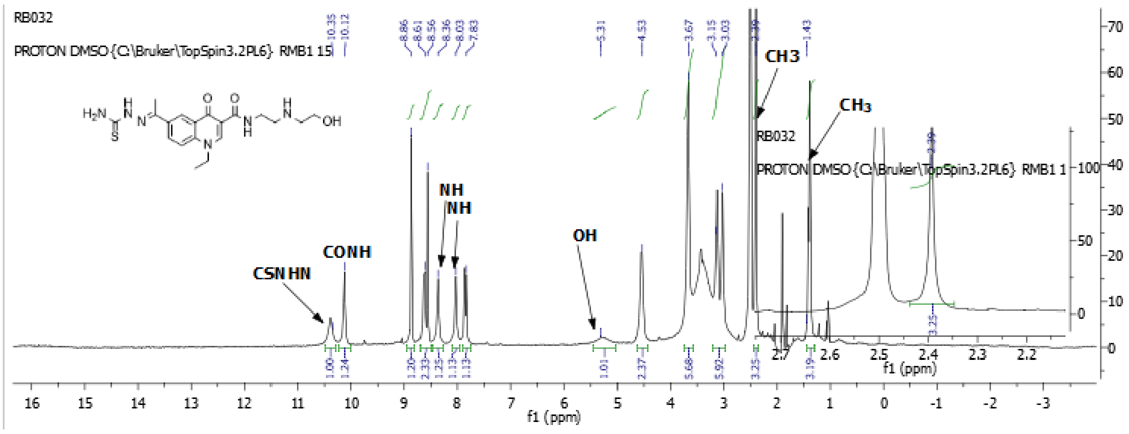
RB032
PROTON DMSO {C:\Bruker\TopSpin3.2PL6} RMB1 15
CSNHHN
CONH
NH
NH
OH
CH3
CH3
f1 (ppm)

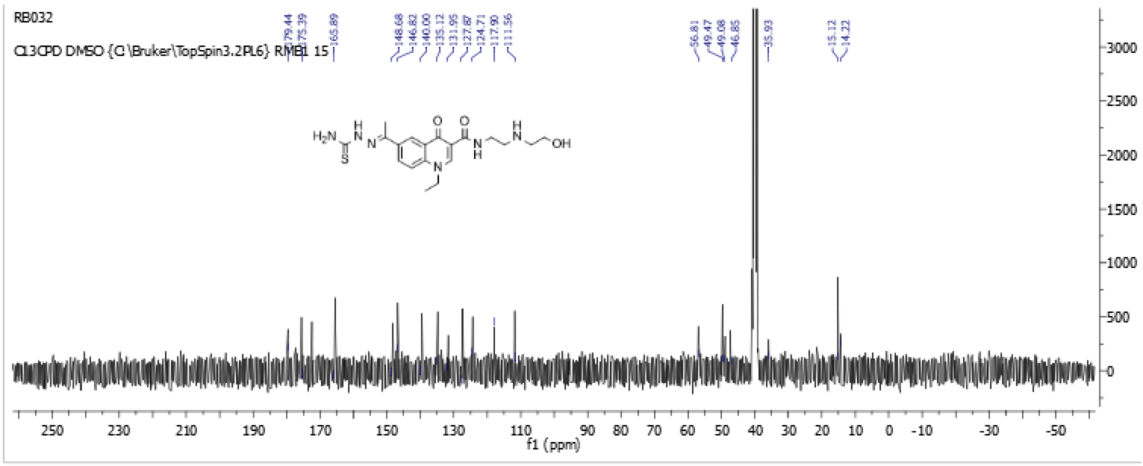
RB032
C13CPD DMSO {C:\Bruker\TopSpin3.2PL6} RMB1 15
f1 (ppm)

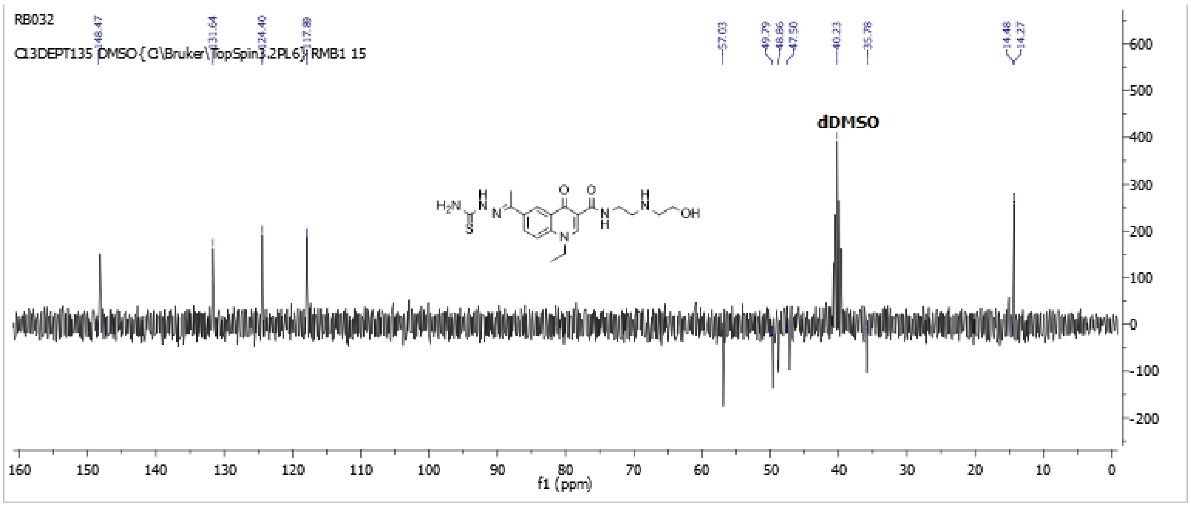
RB032
C13DEPT135 DMSO {C:\Bruker\TopSpin3.2PL6} RMB1 15
dDMSO
f1 (ppm)

COMPOUND 13

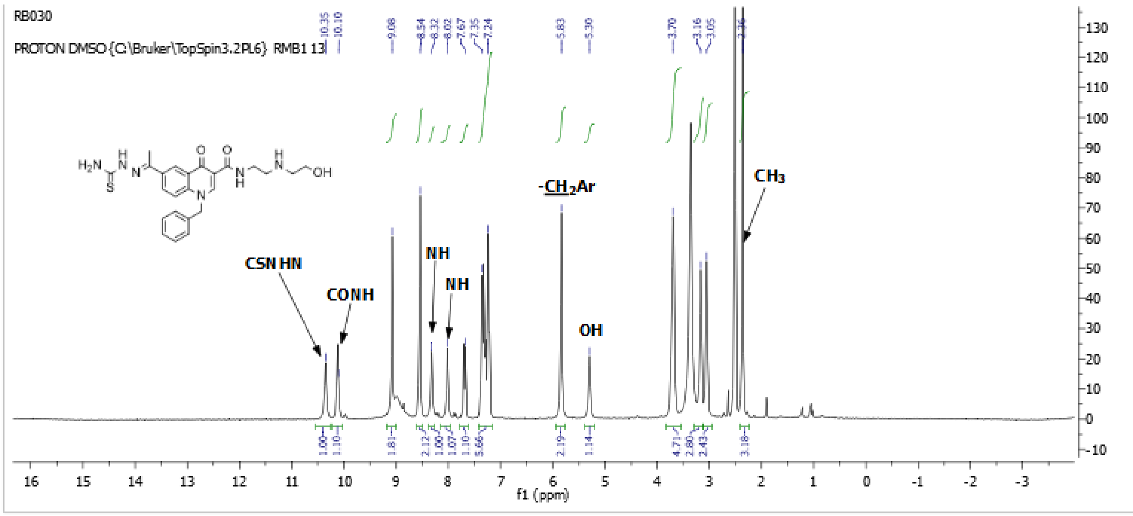
RB030
PROTON DMSO {C:\Bruker\TopSpin3.2PL6} RMB1 13
CSN HN
CONH
NH
NH
-CH2Ar
OH
CH3
f1 (ppm)

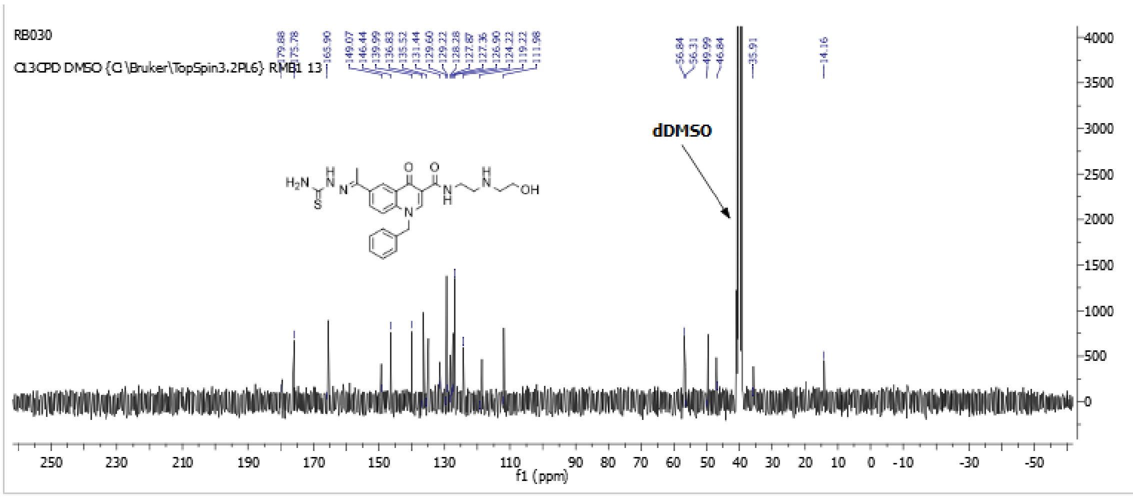
RB030
C13CPD DMSO {C:\Bruker\TopSpin3.2PL6} RMB1 13
dDMSO
f1 (ppm)

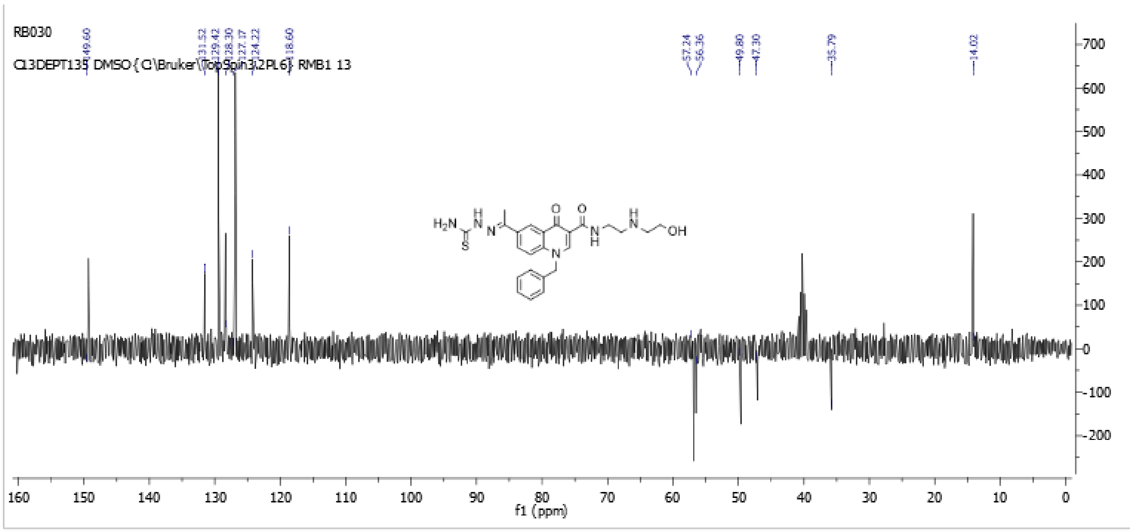
RB030
C13DEPT135 DMSO {C:\Bruker\TopSpin3.2PL6} RMB1 13
f1 (ppm)

COMPOUND 14

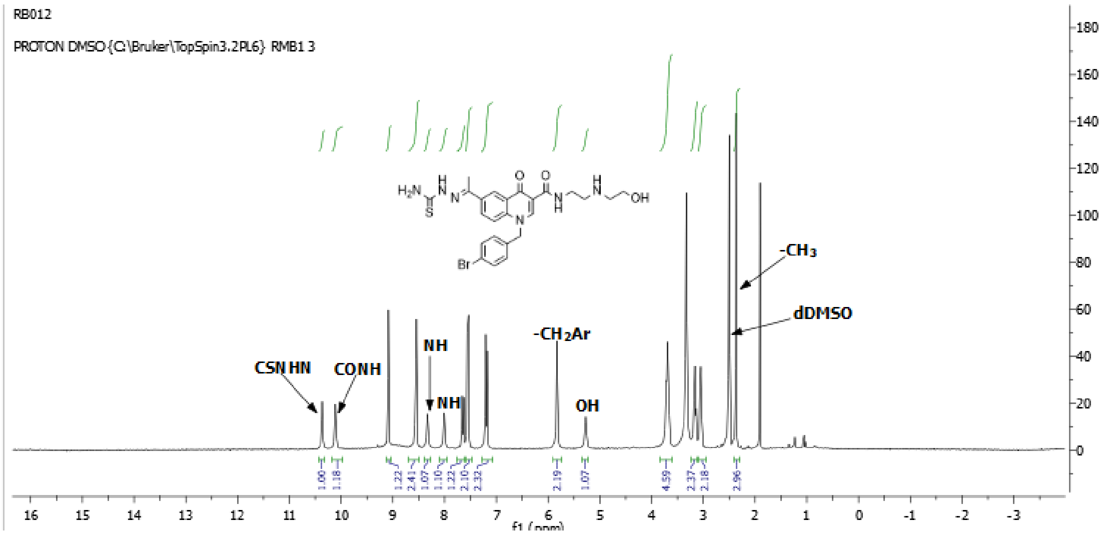
RB012
PROTON DMSO {C:\Bruker\TopSpin3.2PL6} RMB1 3
CSNHHN
CONH
NH
NH
-CH2Ar
OH
-CH3
dDMSO
1.00 1.18 1.22 2.41 1.07 1.10 1.22 2.10 2.32 2.19 1.07 4.59 2.37 2.18 2.96
f1 (ppm)

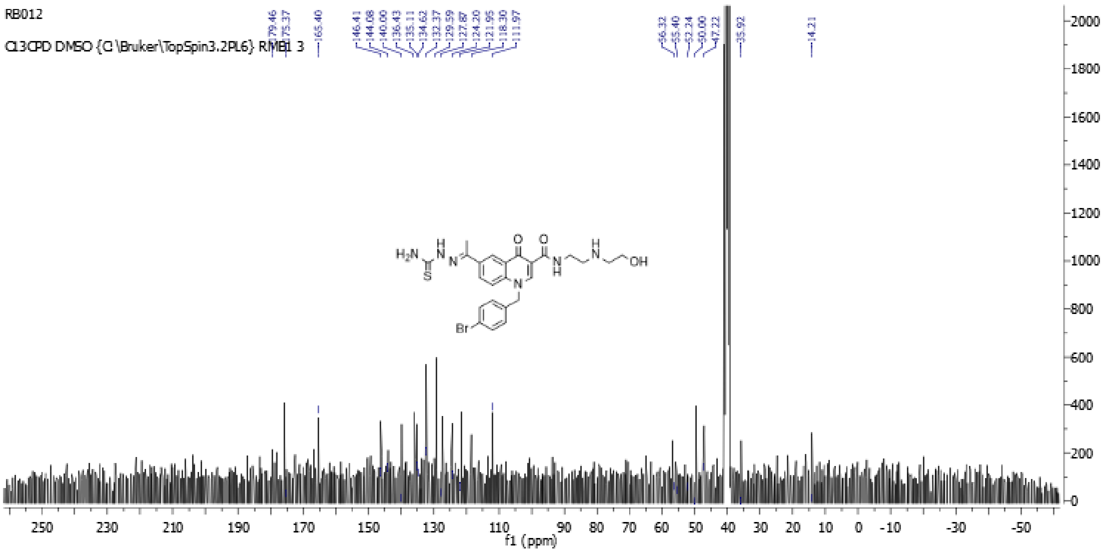
RB012
C13CPD DMSO {C:\Bruker\TopSpin3.2PL6} RMB1 3
179.46 175.37 165.40 146.41 144.08 140.00 136.43 135.11 134.62 132.37 129.59 127.87 124.20 121.95 118.30 111.97 56.32 55.40 52.24 50.00 47.22 35.92 14.21
f1 (ppm)

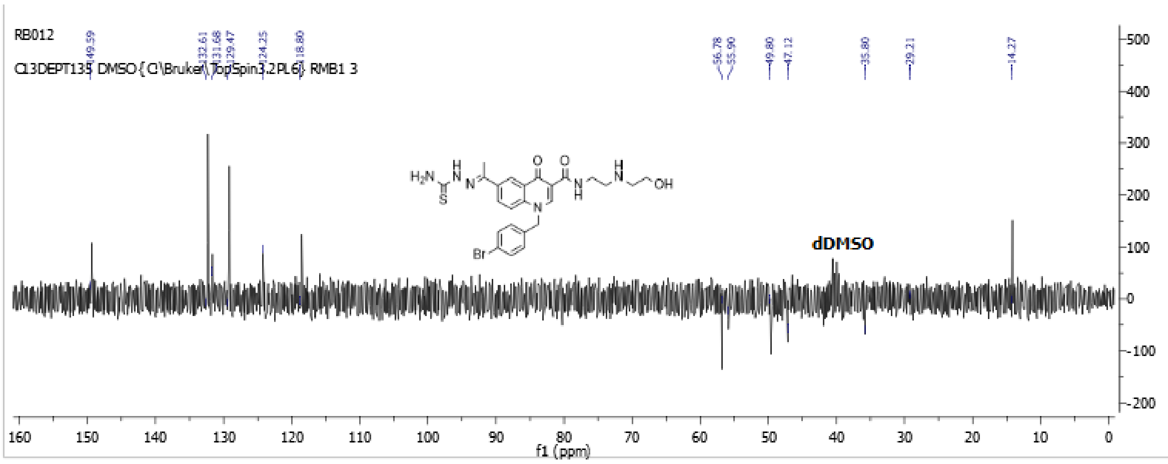
RB012
C13DEPT135 DMSO {C:\Bruker\TopSpin3.2PL6} RMB1 3
149.59 132.61 131.68 129.47 124.25 118.80 56.78 55.90 49.80 47.12 35.80 29.21 14.27
dDMSO
f1 (ppm)

COMPOUND 15

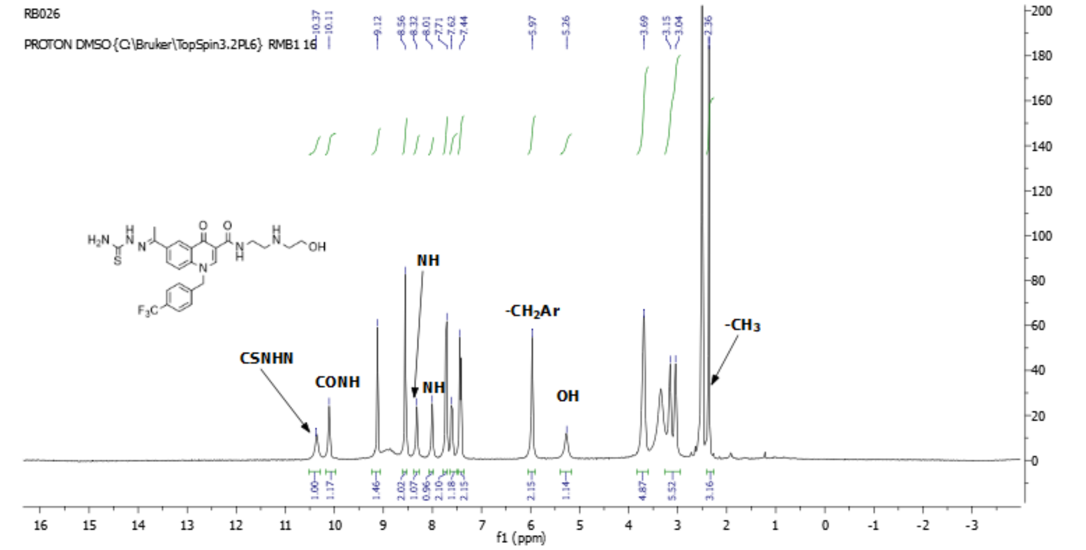

RB026
PROTON DMSO {C:\Bruker\TopSpin3.2PL6} RMB1 16
CSNHN
CONH
NH
NH
-CH2Ar
OH
-CH3
f1 (ppm)

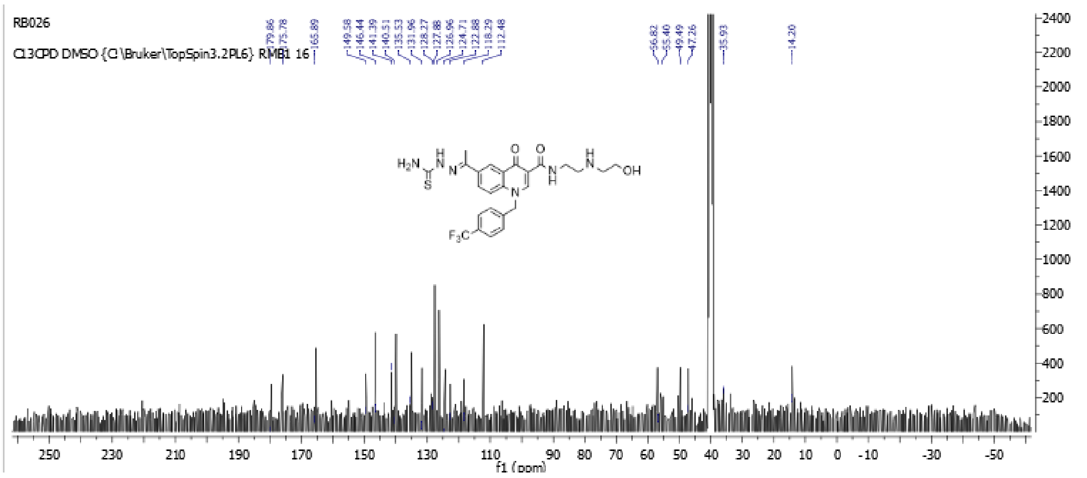

RB026
C13CPD DMSO {C:\Bruker\TopSpin3.2PL6} RMB1 16
f1 (ppm)

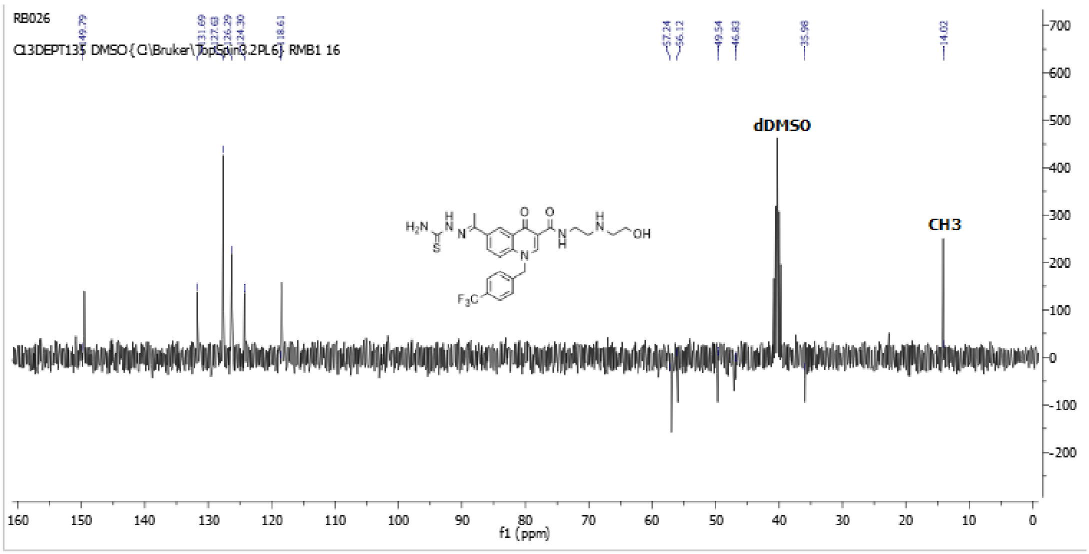

RB026
C13DEPT135 DMSO {C:\Bruker\TopSpin3.2PL6} RMB1 16
dDMSO
CH3
f1 (ppm)

COMPOUND 16

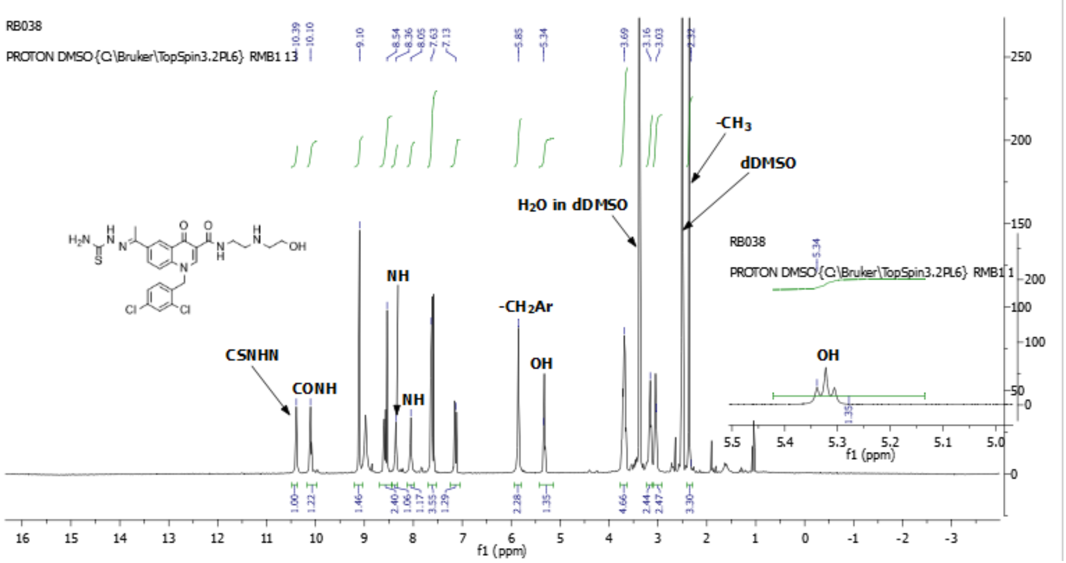
RB038
PROTON DMSO {C:\Bruker\TopSpin3.2PL6} RMB1 13
10.39
10.10
9.10
8.54
8.36
8.05
7.63
7.13
5.85
5.34
3.69
3.16
3.03
2.32
-CH$_3$
dDMSO
H$_2$O in dDMSO
NH
-CH$_2$Ar
CSNHN
CONH
NH
OH
OH
f1 (ppm)

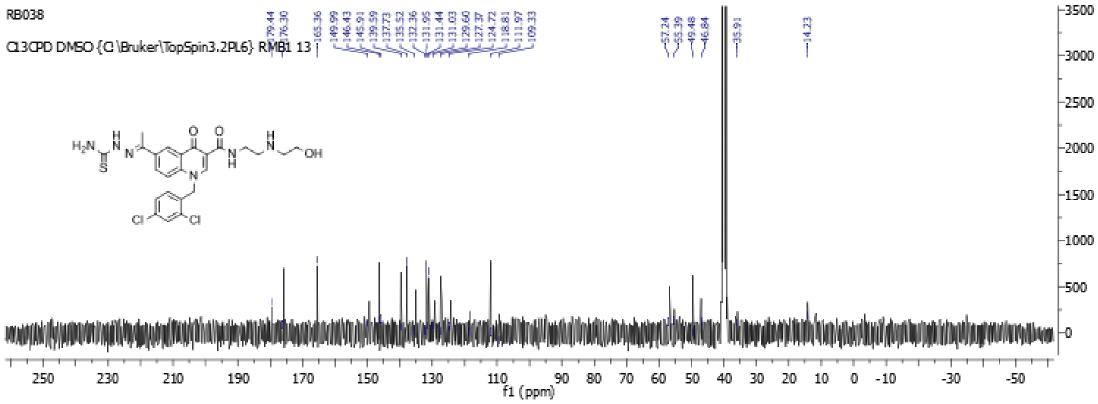
RB038
C13CPD DMSO {C:\Bruker\TopSpin3.2PL6} RMB1 13
f1 (ppm)

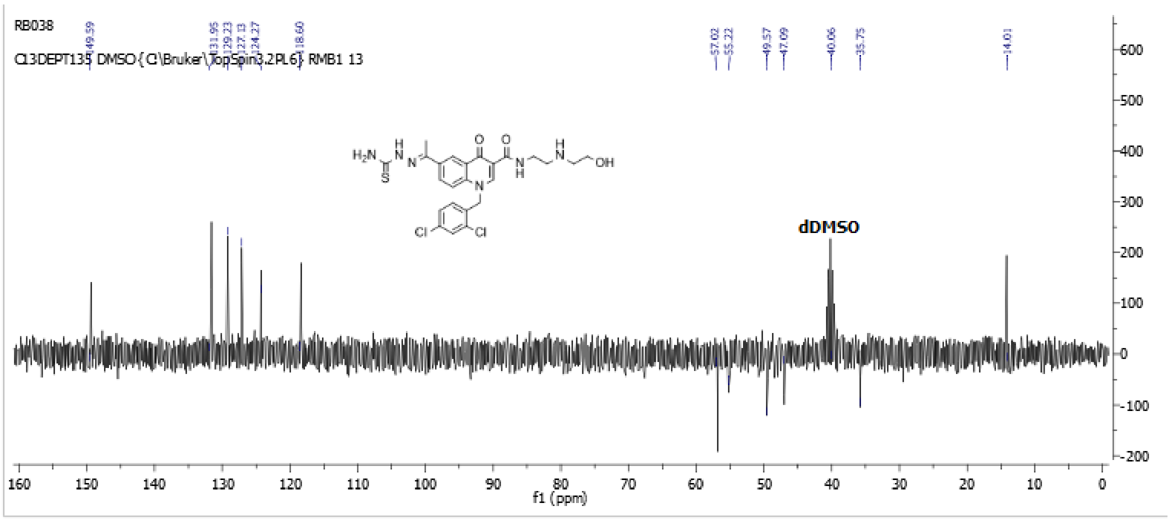
RB038
C13DEPT135 DMSO {C:\Bruker\TopSpin3.2PL6} RMB1 13
dDMSO
f1 (ppm)

COMPOUND 17

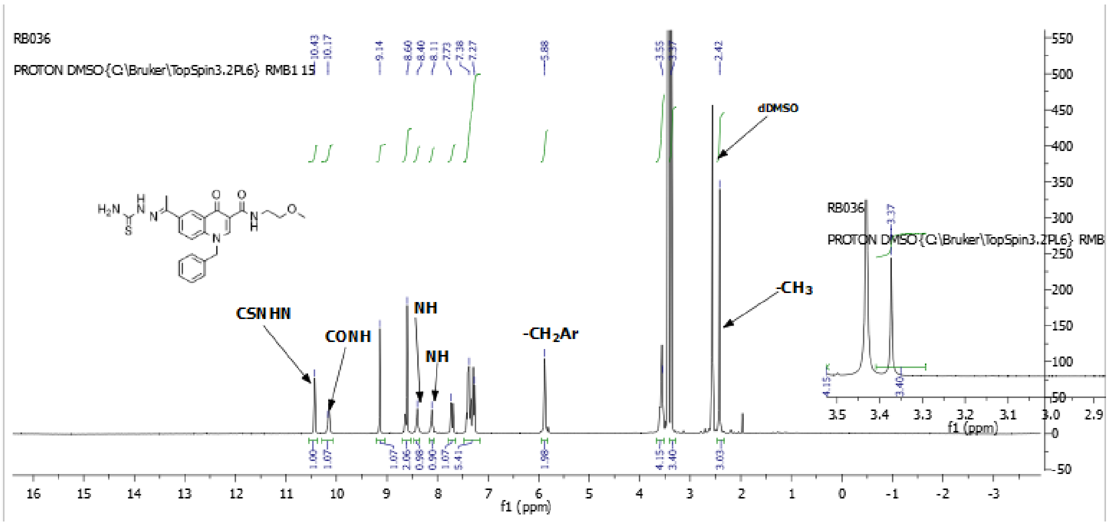
RB036
PROTON DMSO {C:\Bruker\TopSpin3.2PL6} RMB1 15
CSNHHN
CONH
NH
NH
-CH2Ar
dDMSO
-CH3
f1 (ppm)

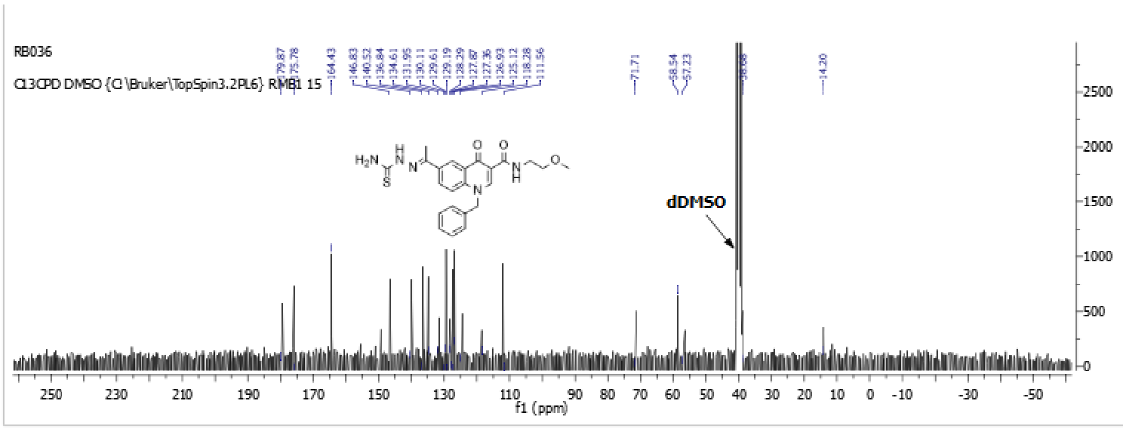
RB036
C13CPD DMSO {C:\Bruker\TopSpin3.2PL6} RMB1 15
dDMSO
f1 (ppm)

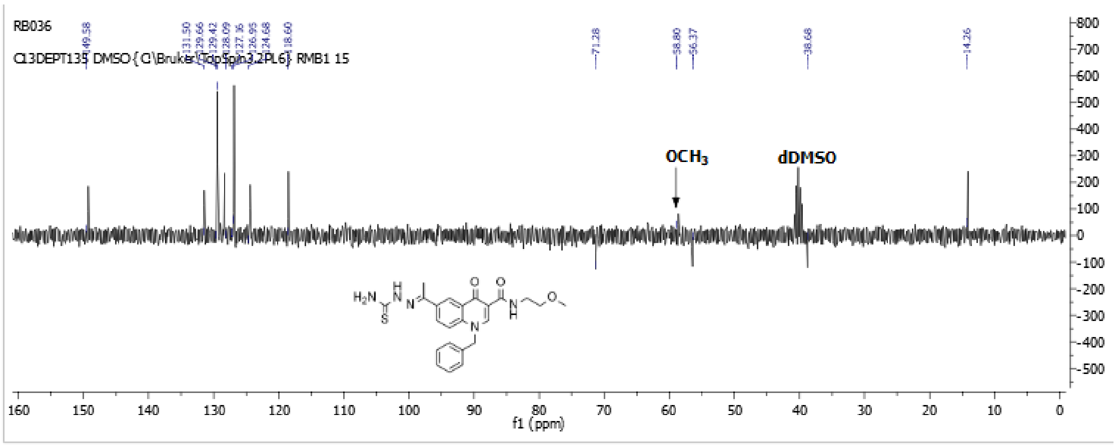
RB036
C13DEPT135 DMSO {C:\Bruker\TopSpin3.2PL6} RMB1 15
OCH3
dDMSO
f1 (ppm)

COMPOUND 18

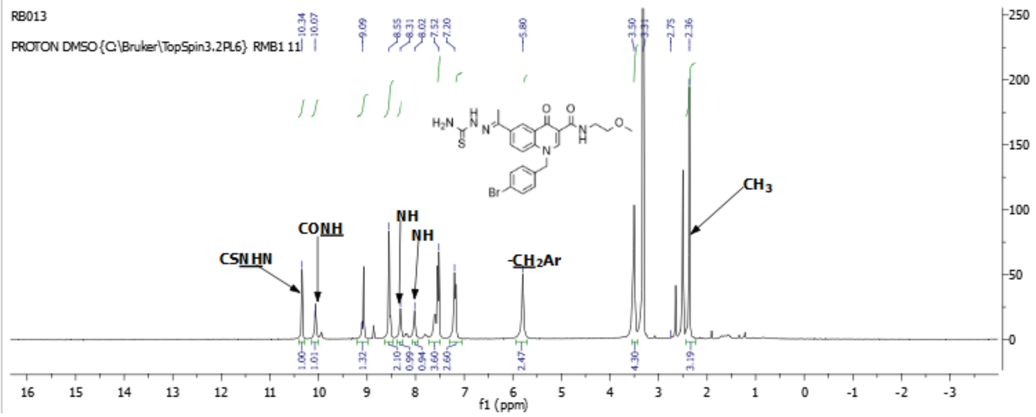
RB013
PROTON DMSO {C:\Bruker\TopSpin3.2PL6} RMB1 11
CSNHN
CONH
NH
NH
-CH2Ar
CH3
f1 (ppm)

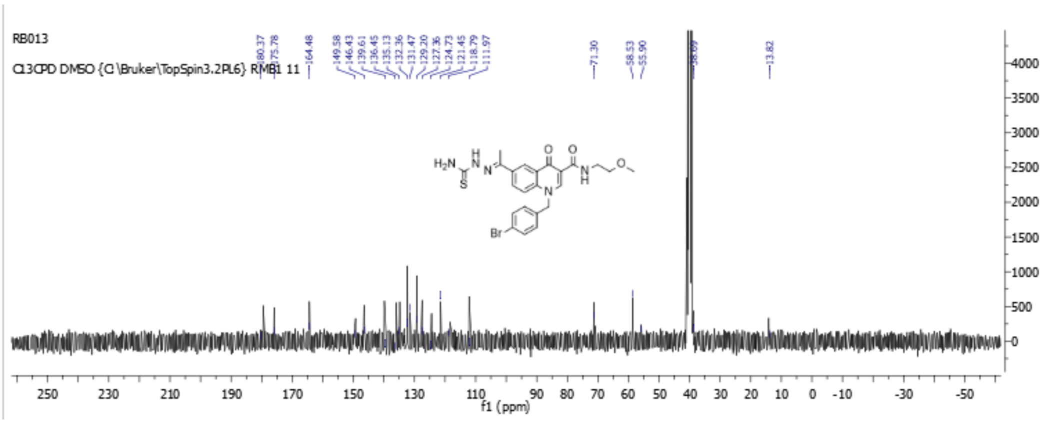
RB013
C13CPD DMSO {C:\Bruker\TopSpin3.2PL6} RMB1 11
f1 (ppm)

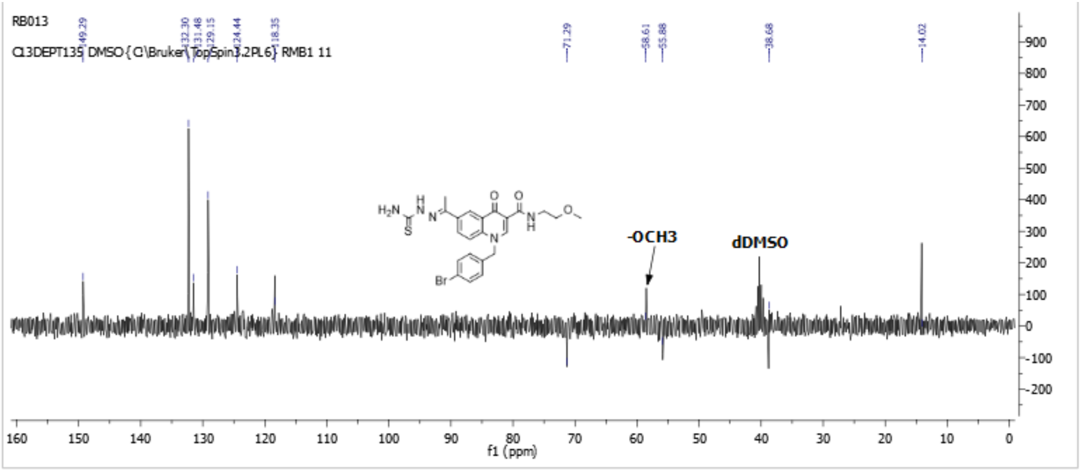
RB013
C13DEPT135 DMSO {C:\Bruker\TopSpin3.2PL6} RMB1 11
-OCH3
dDMSO
f1 (ppm)

COMPOUND 19

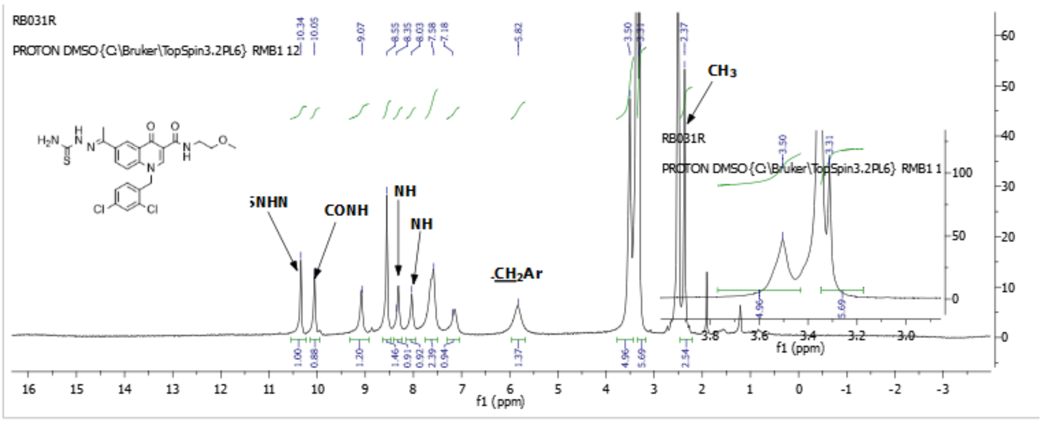
RB031R
PROTON DMSO {C:\Bruker\TopSpin3.2PL6} RMB1 12
SNHN
CONH
NH
NH
CH2Ar
CH3
f1 (ppm)

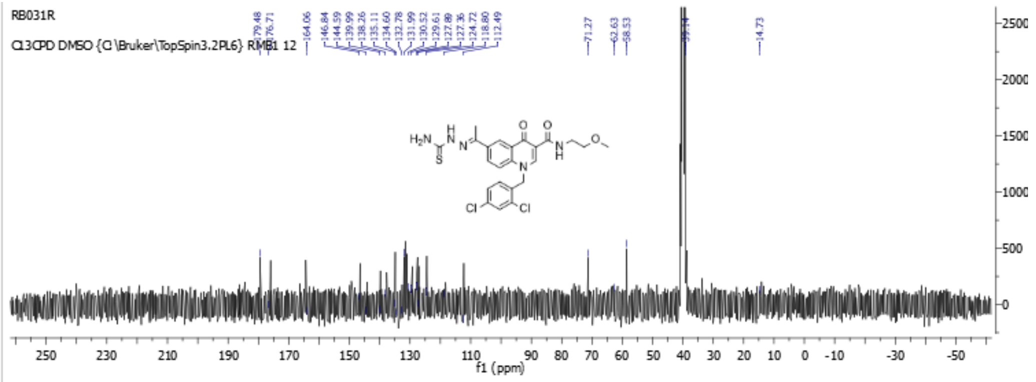
RB031R
C13CPD DMSO {C:\Bruker\TopSpin3.2PL6} RMB1 12
f1 (ppm)

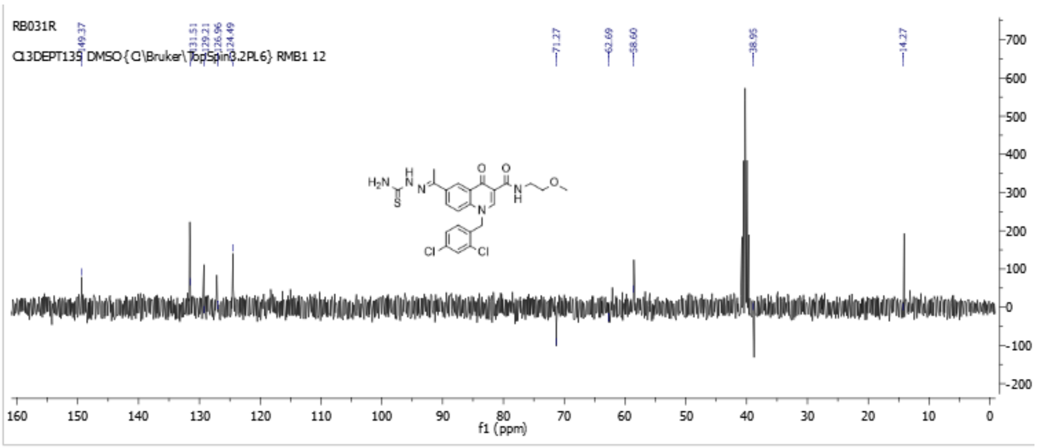
RB031R
C13DEPT135 DMSO {C:\Bruker\TopSpin3.2PL6} RMB1 12
f1 (ppm)

COMPOUND 20

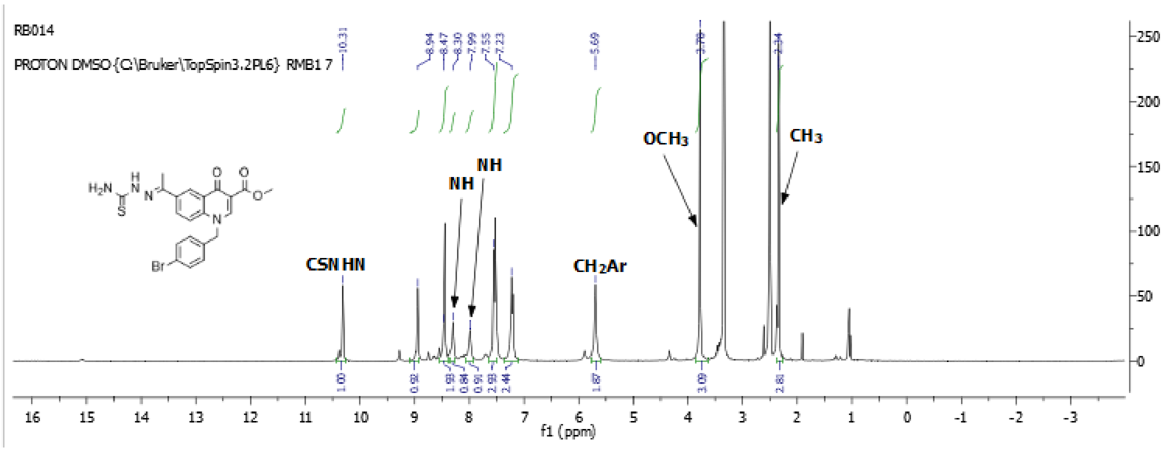
RB014
PROTON DMSO {C:\Bruker\TopSpin3.2PL6} RMB1 7
CSN HN
NH
NH
CH2Ar
OCH3
CH3
f1 (ppm)

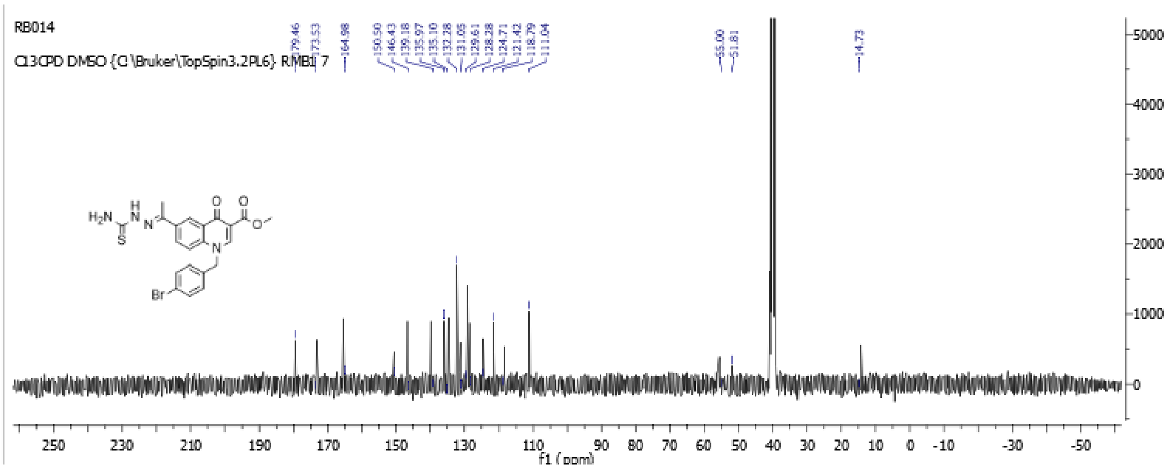
RB014
C13CPD DMSO {C:\Bruker\TopSpin3.2PL6} RMB1 7
f1 (ppm)

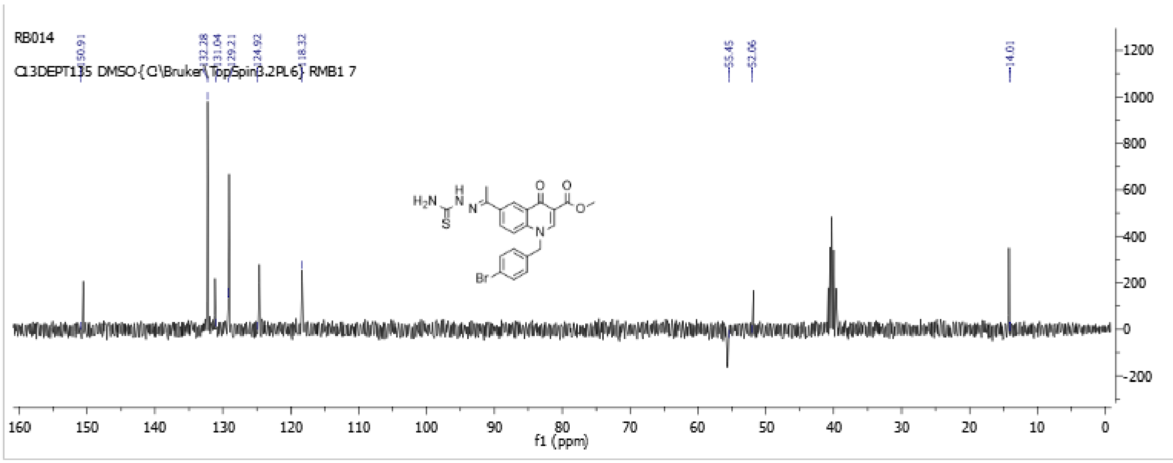
RB014
C13DEPT135 DMSO {C:\Bruker\TopSpin3.2PL6} RMB1 7
f1 (ppm)

COMPOUND 21

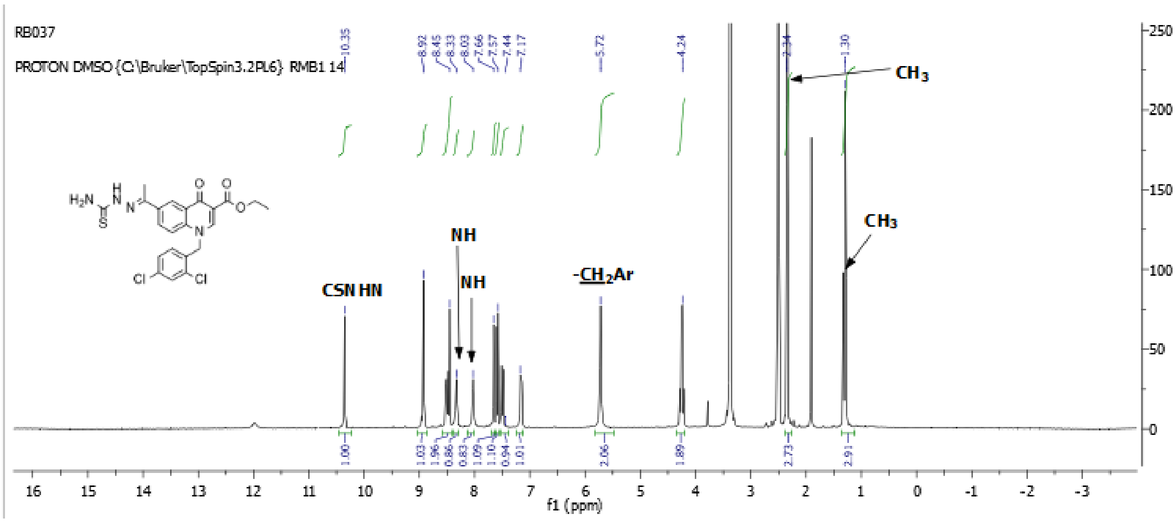
RB037
PROTON DMSO {C:\Bruker\TopSpin3.2PL6} RMB1 14
10.35
8.92
8.45
8.33
8.03
7.66
7.57
7.44
7.17
5.72
4.24
2.34
1.30
CH3
CH3
NH
NH
CSN HN
-CH2Ar
1.00
1.03
1.96
0.86
0.83
1.09
1.10
0.94
1.01
2.06
1.89
2.73
2.91
f1 (ppm)

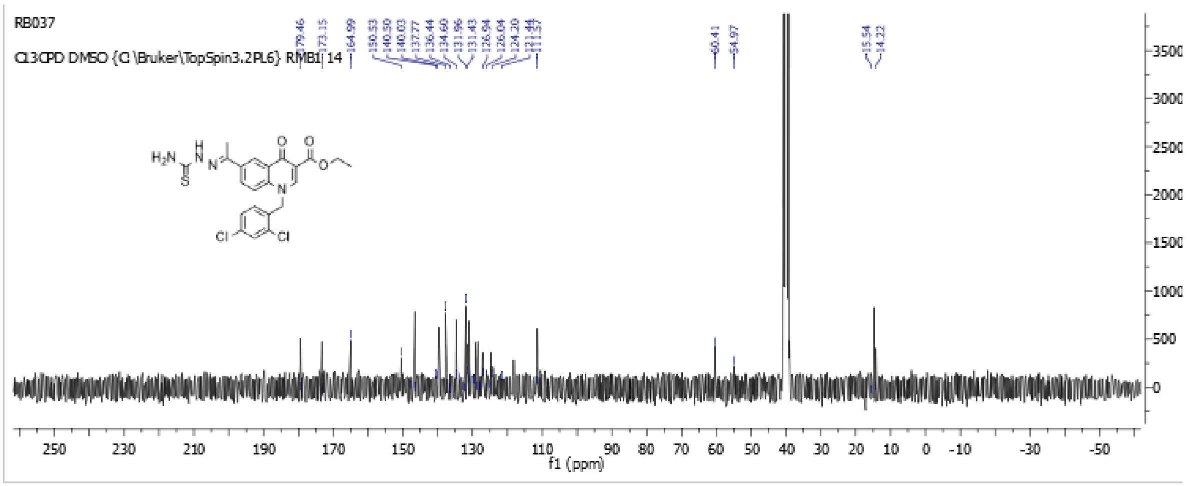
RB037
C13CPD DMSO {C:\Bruker\TopSpin3.2PL6} RMB1 14
179.46
173.15
164.99
150.53
140.50
140.03
137.77
136.44
134.60
131.96
131.43
126.94
126.04
124.20
121.44
111.57
60.41
54.97
15.54
14.22
f1 (ppm)

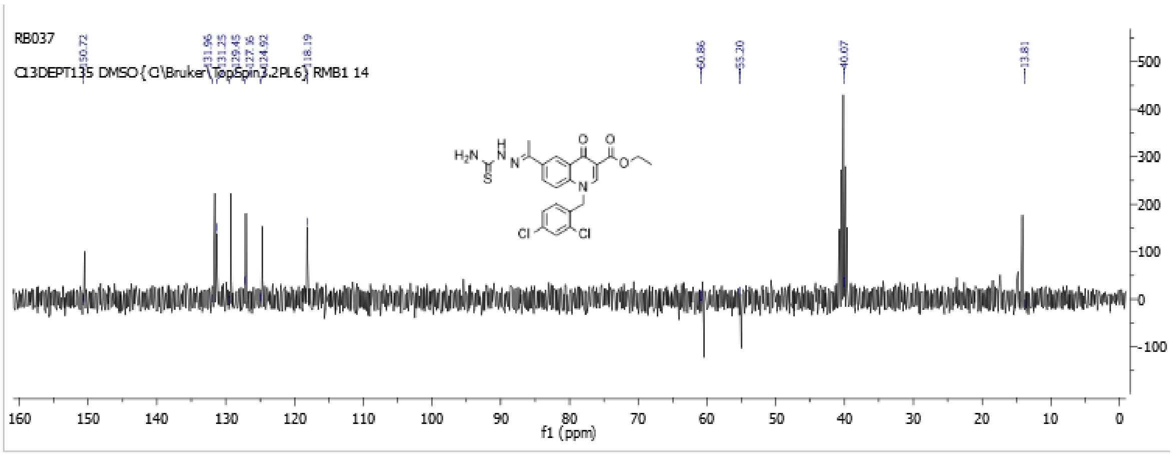
RB037
C13DEPT135 DMSO {C:\Bruker\TopSpin3.2PL6} RMB1 14
150.72
131.96
131.25
129.45
127.36
124.92
118.19
60.86
55.20
40.07
13.81
f1 (ppm)

COMPOUND 11

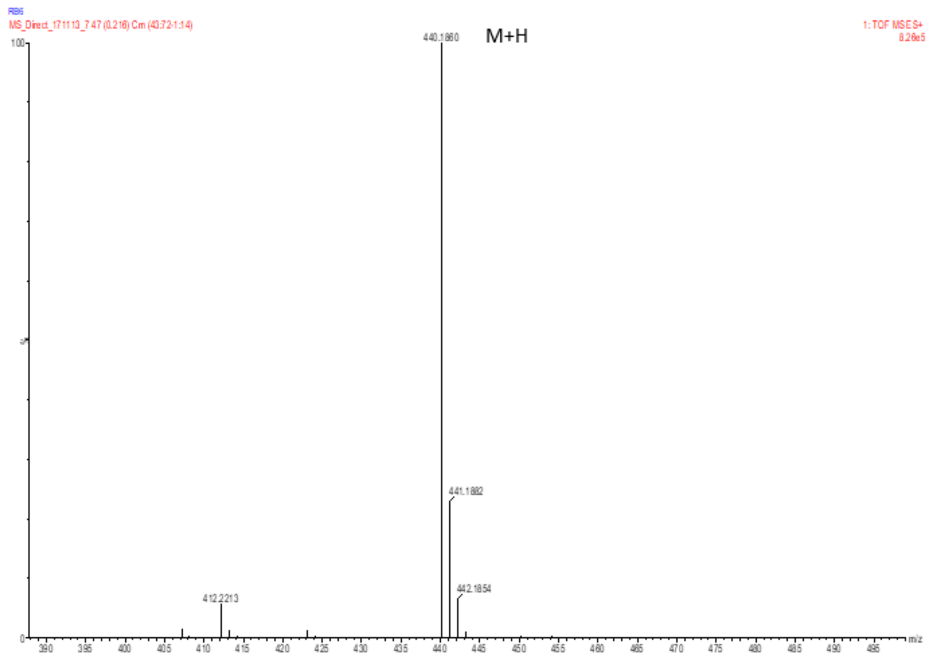

COMPOUND 12

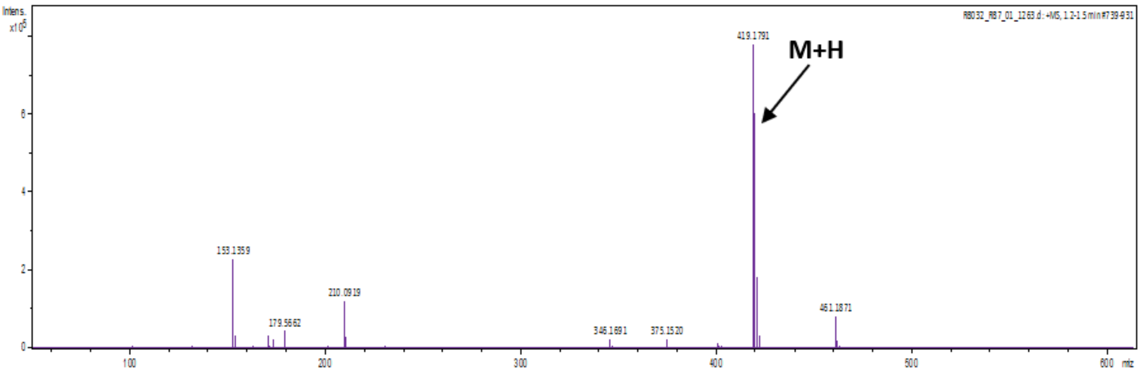

COMPOUND 13

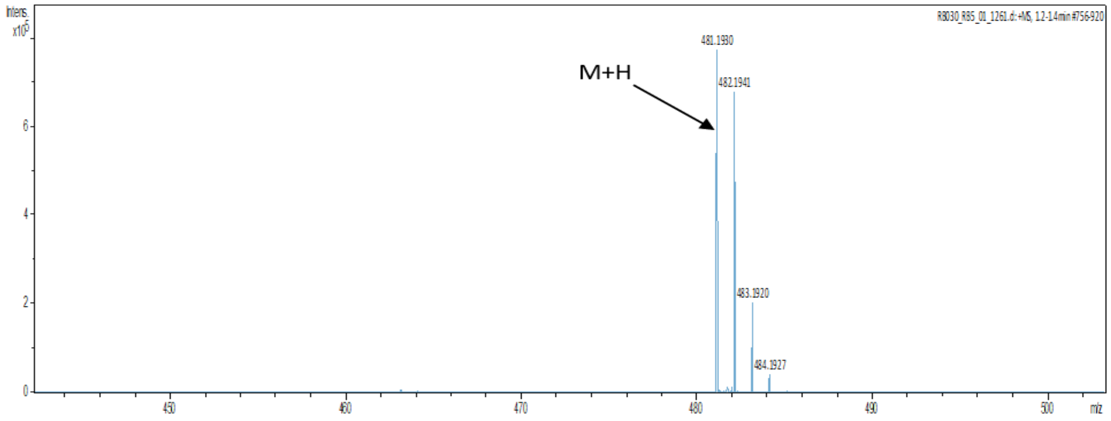

COMPOUND 14

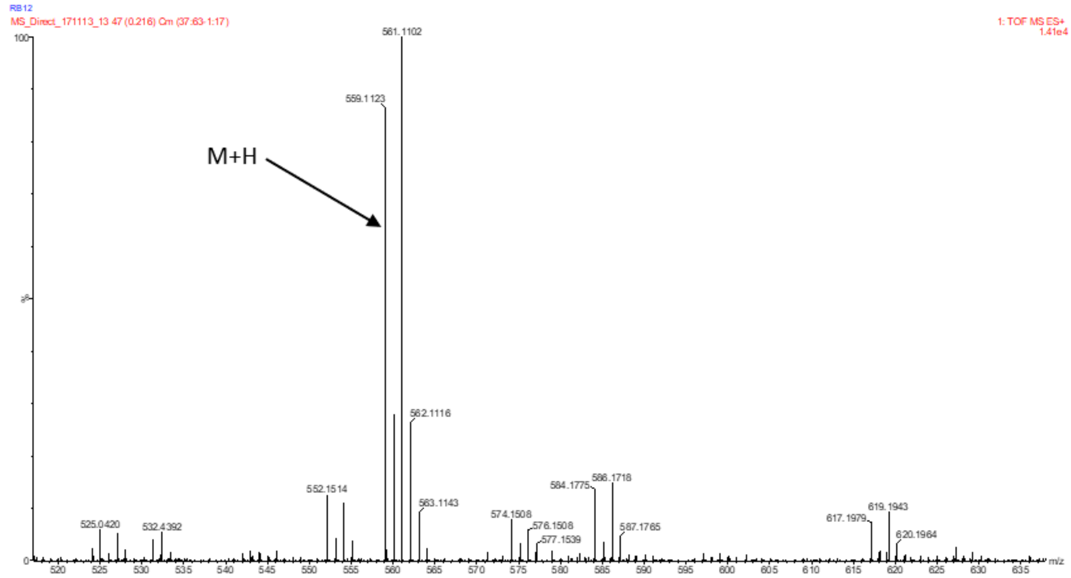

COMPOUND 15

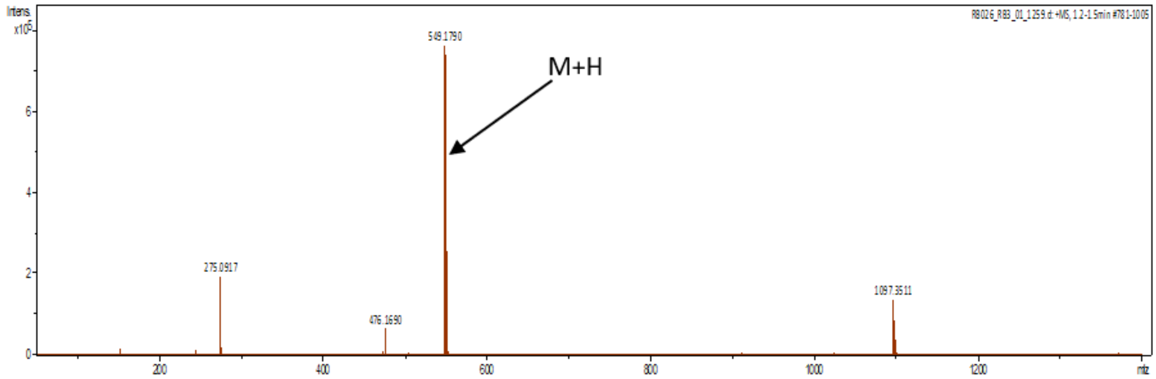

COMPOUND 16

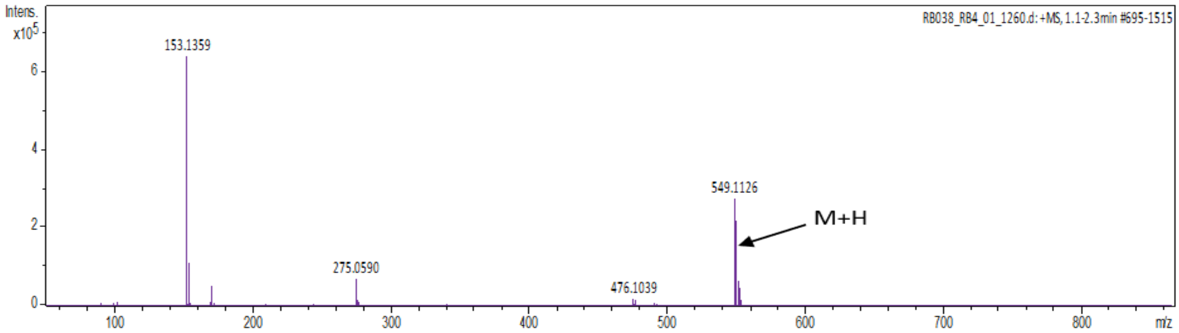

COMPOUND 17

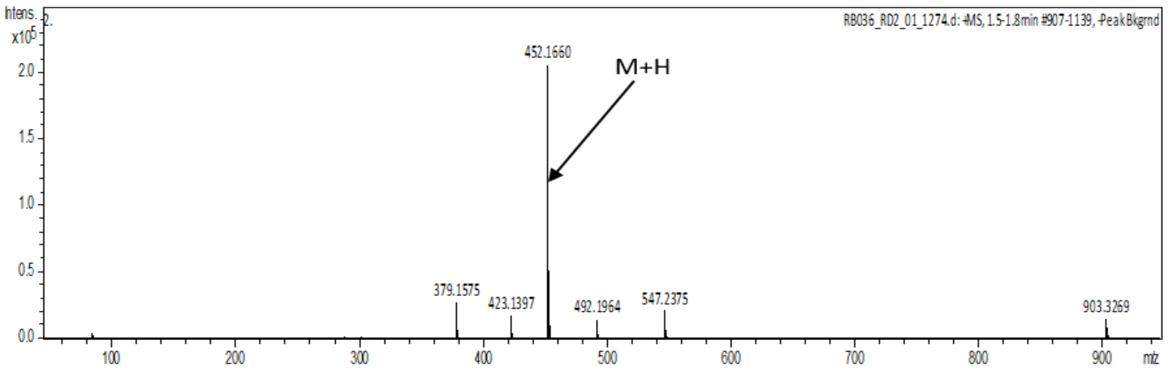

COMPOUND 18

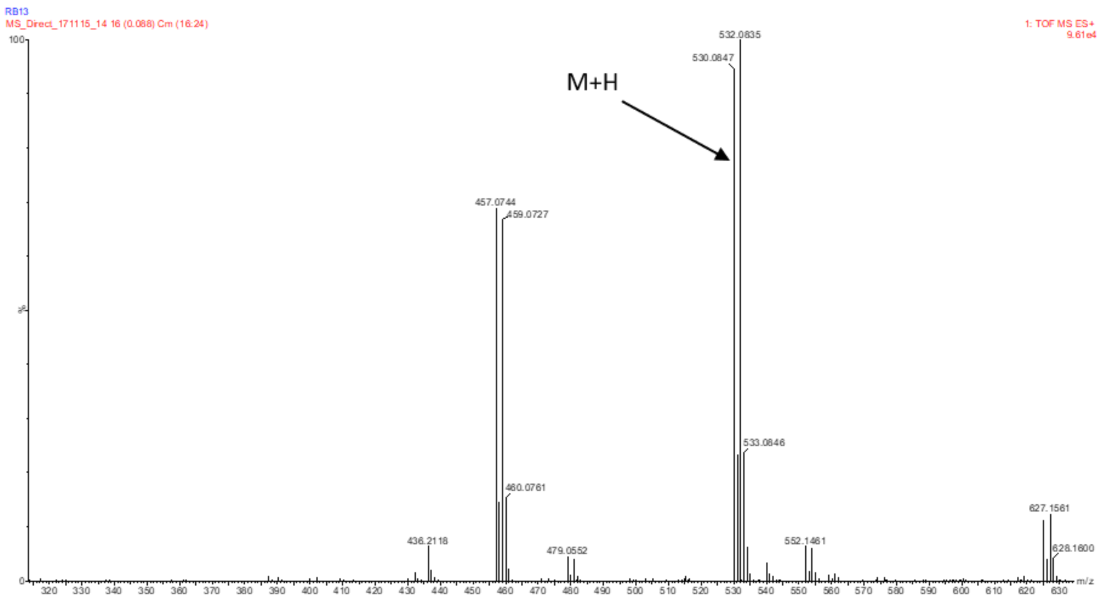

COMPOUND 19

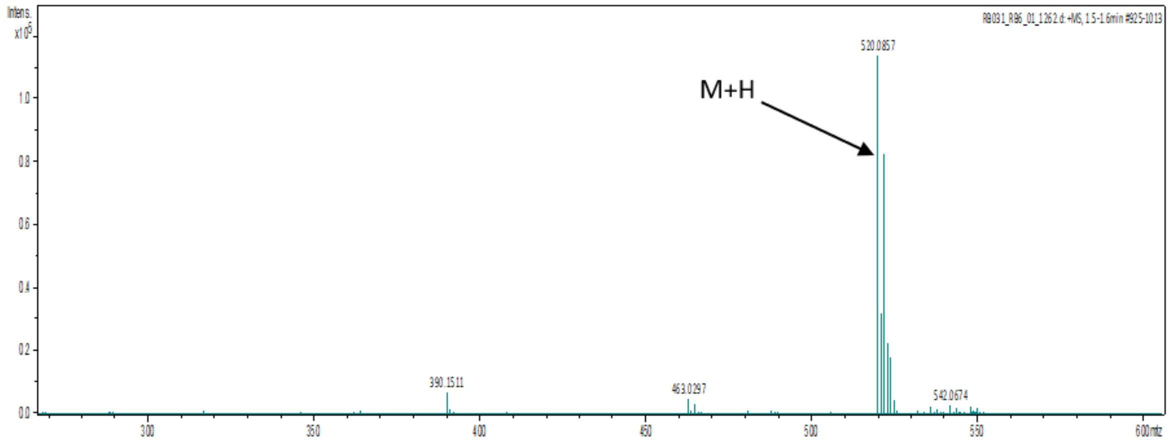

COMPOUND 20

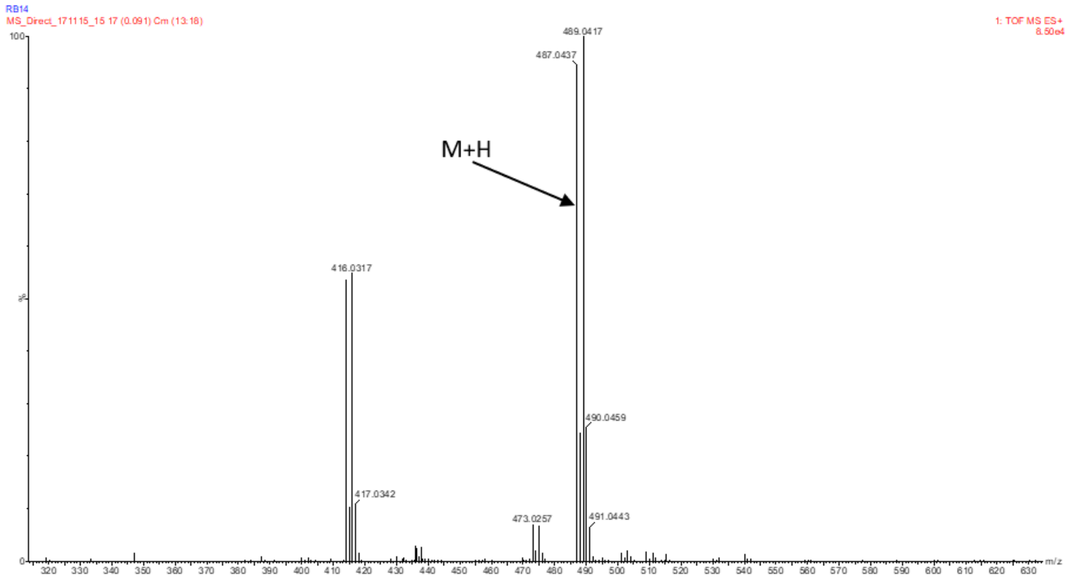

COMPOUND 21

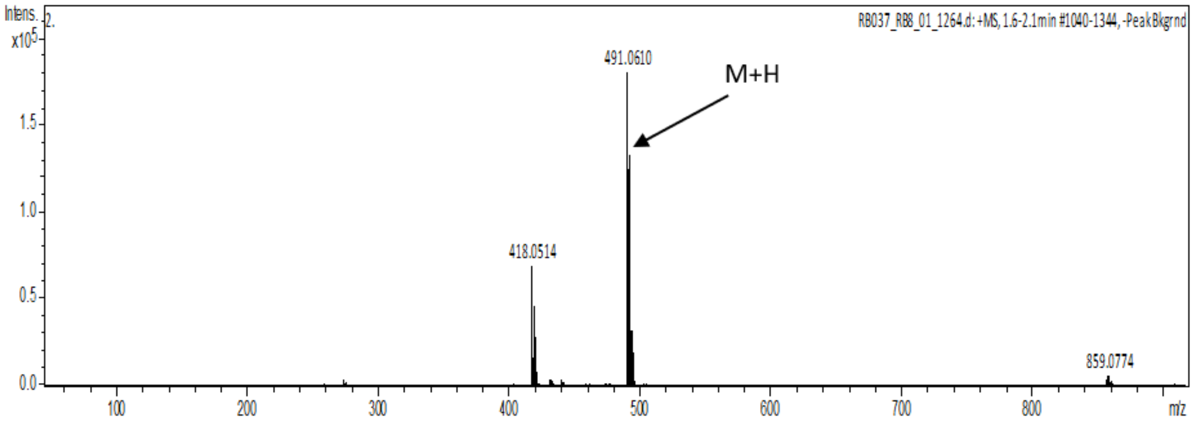

COMPOUND 11

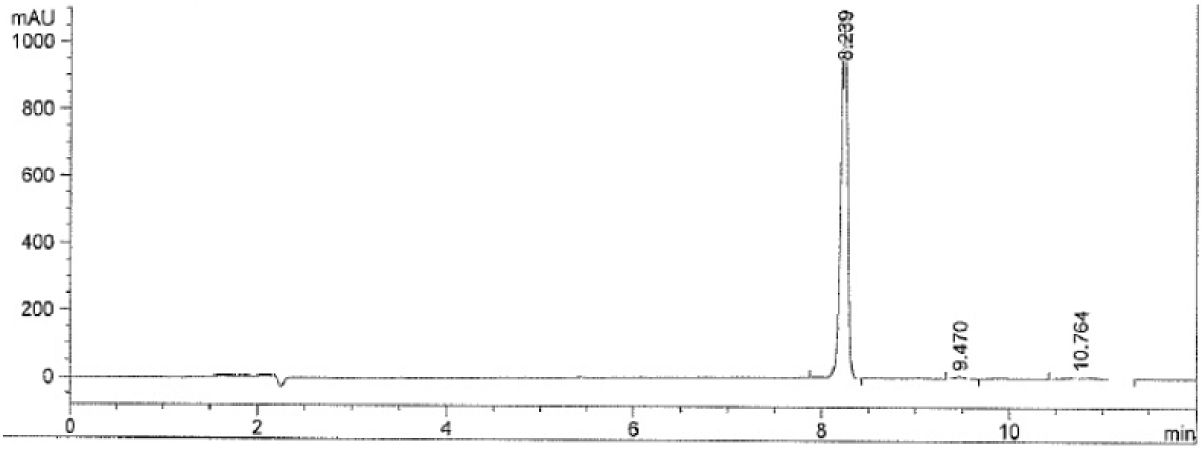

COMPOUND 12

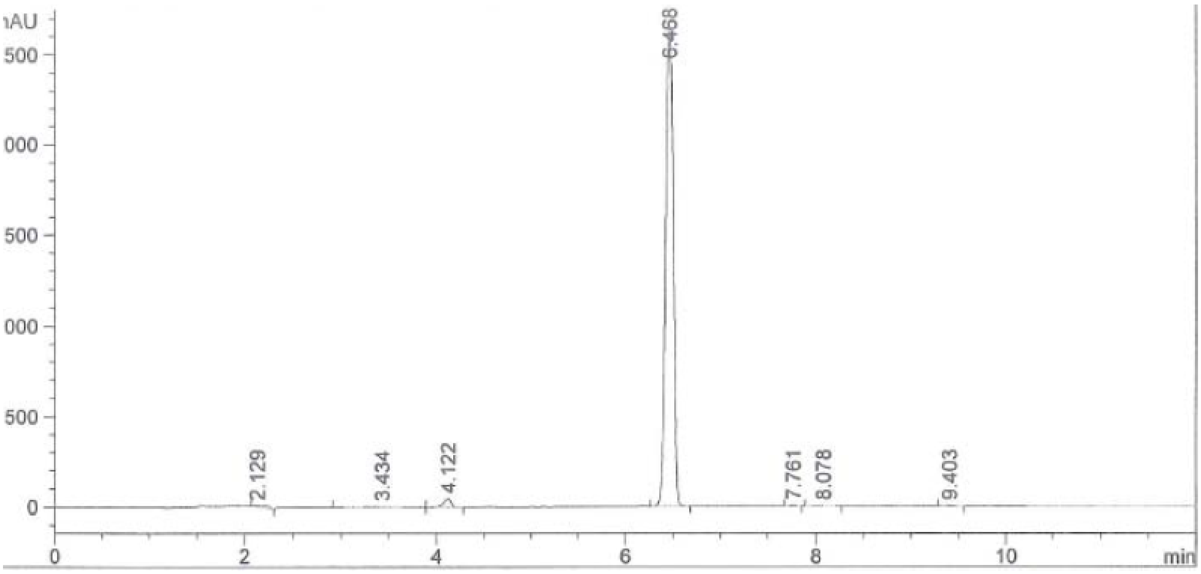

COMPOUND 13

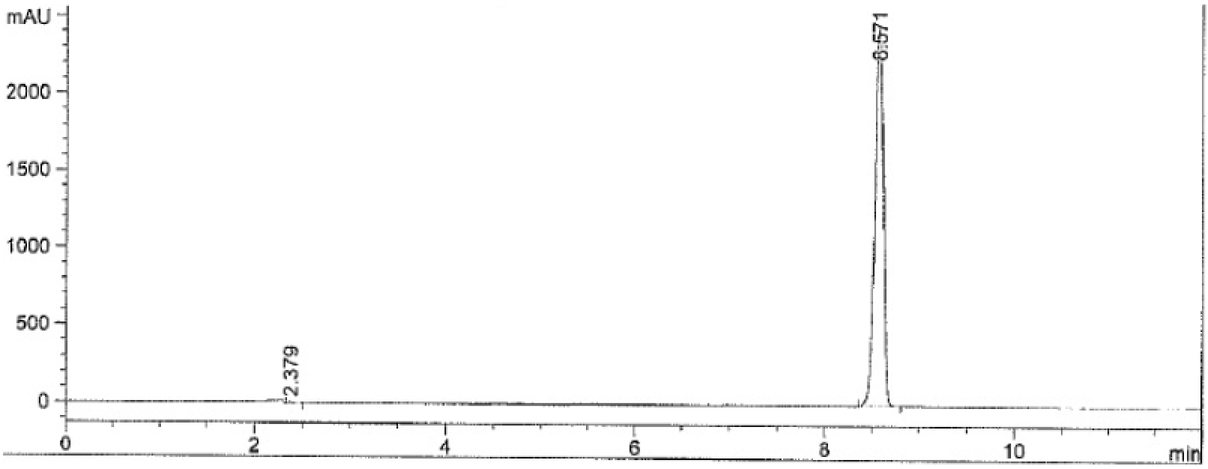

COMPOUND 14

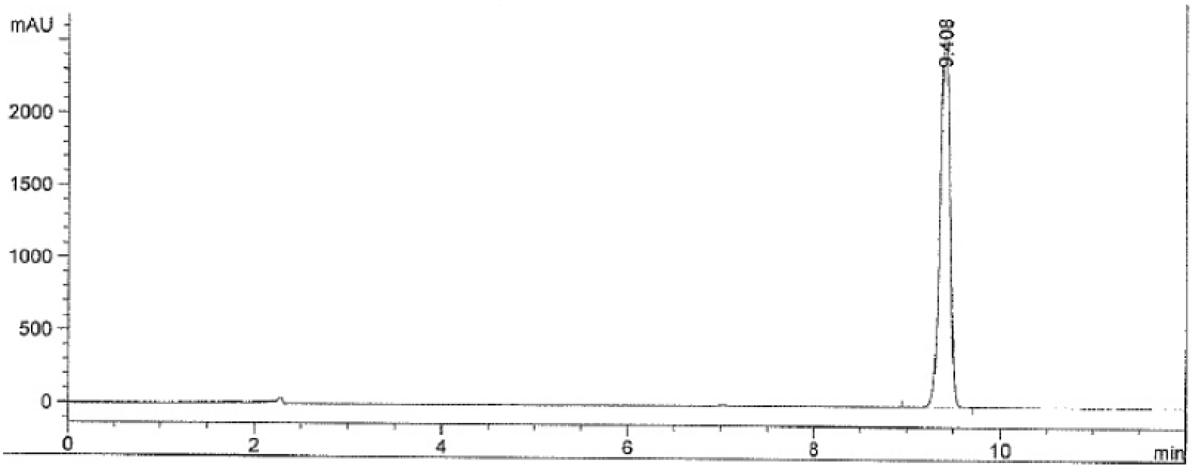

COMPOUND 15

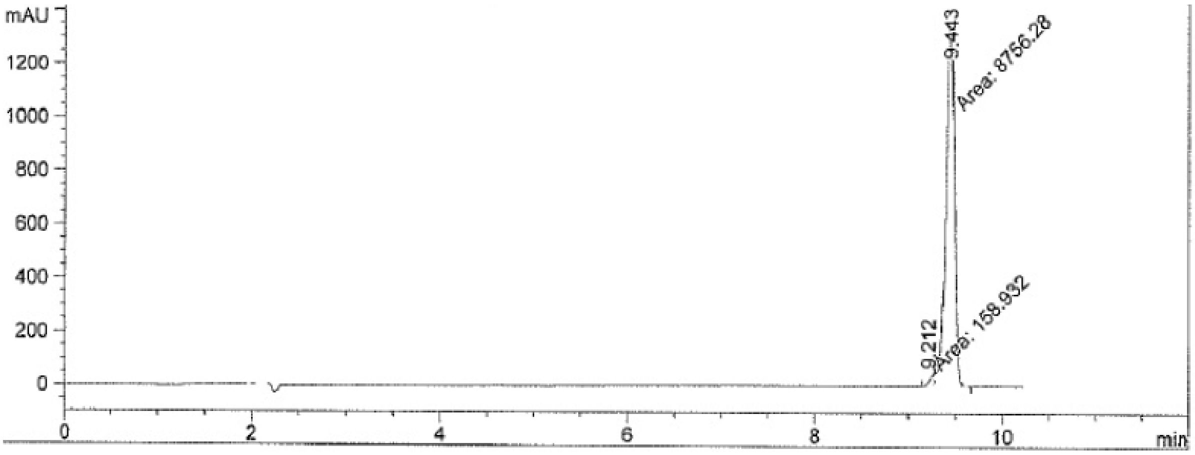

COMPOUND 16

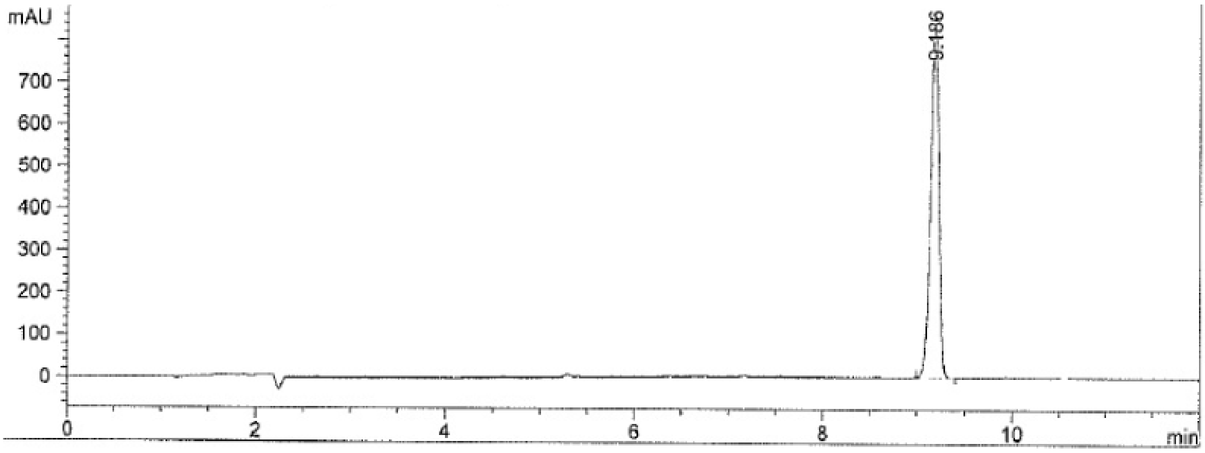

COMPOUND 17

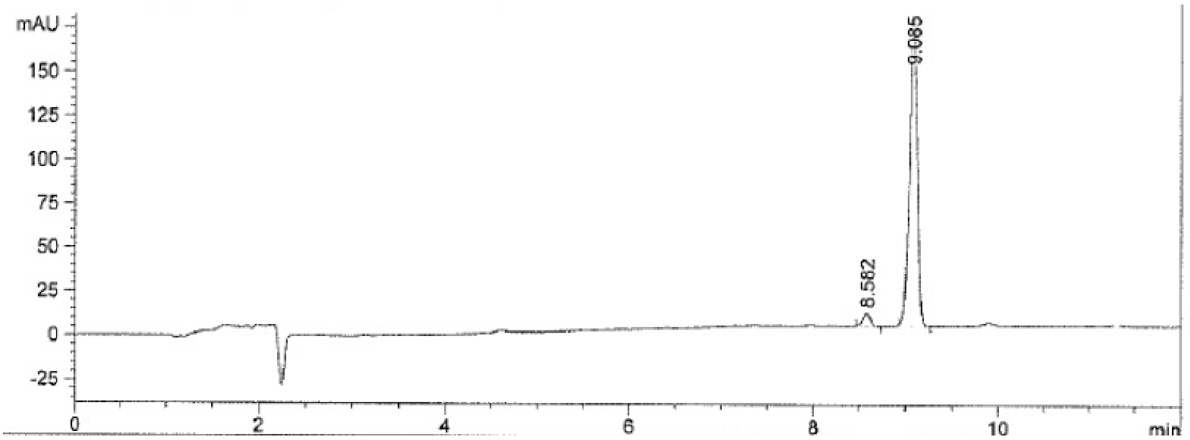

COMPOUND 18

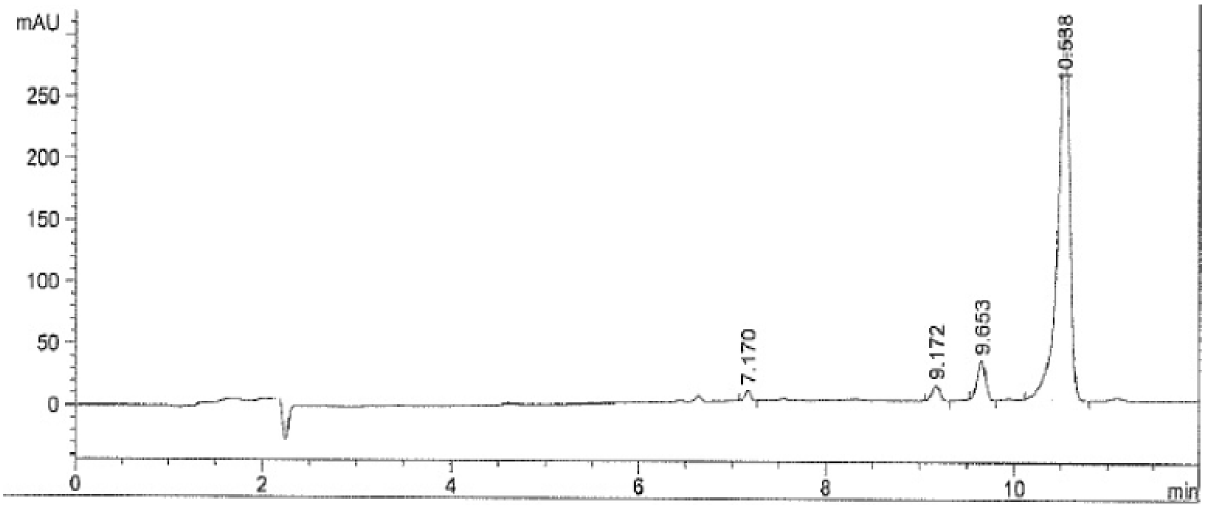

COMPOUND 19

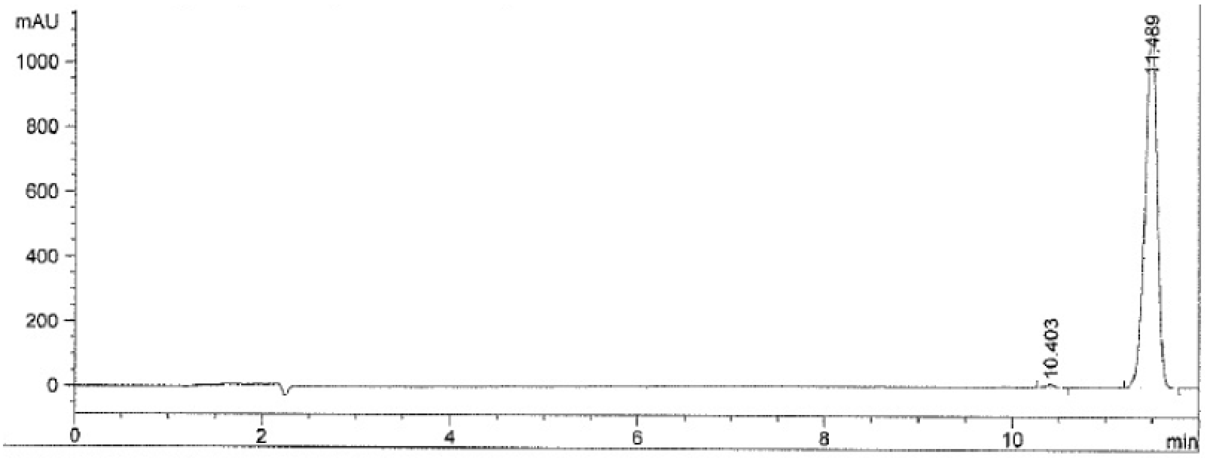

COMPOUND 20

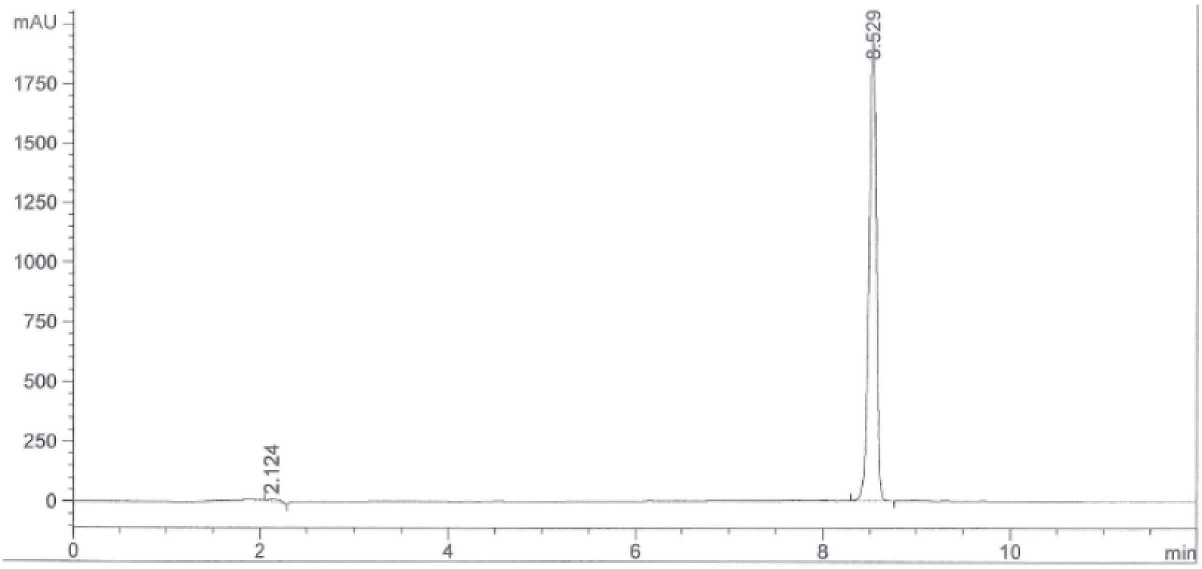

COMPOUND 21

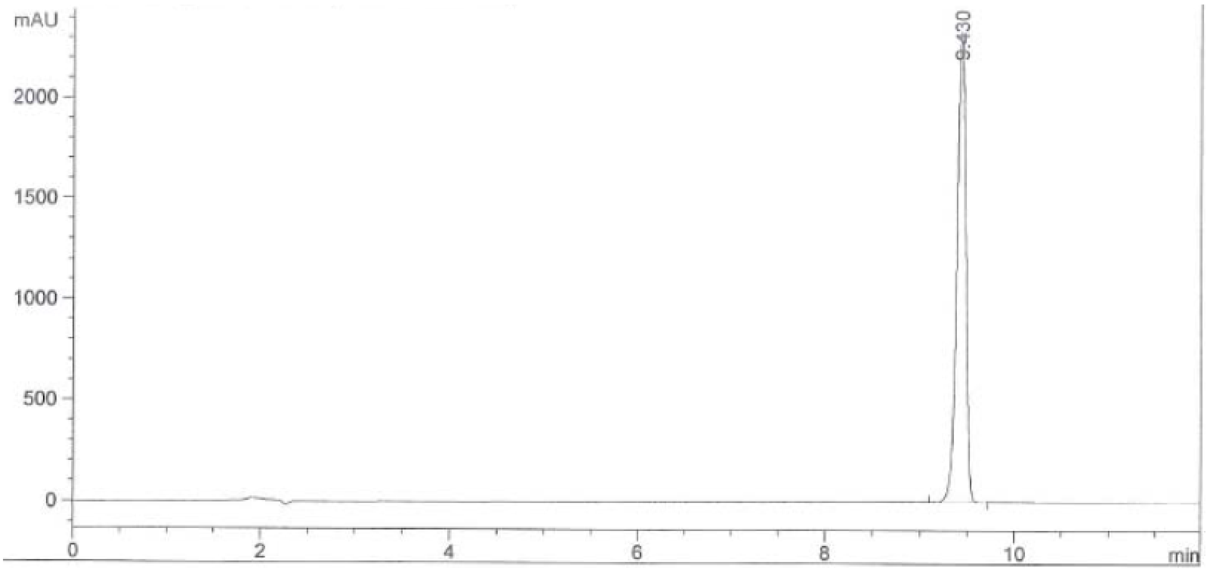

Supplement: Supplementary file 1 [file molecules-24-01740-s001.pdf]
